# Current status of immunotherapy for non-small cell lung cancer

**DOI:** 10.3389/fphar.2022.989461

**Published:** 2022-10-13

**Authors:** Tao Yang, Yilin Xiong, Yufei Zeng, Yan Wang, Jing Zeng, Jie Liu, Shangfu Xu, Li-Sheng Li

**Affiliations:** Zunyi Medical University, Zunyi, China

**Keywords:** non-small cell lung cancer, immunotherapy, antibody-drug conjugates, immune checkpoint inhibitor, bispecific antibodies, TCR-T and CAR-T therapy

## Abstract

Nowadays, lung cancer is still the deadliest oncological disease in the world. Among them, non-small cell lung cancer (NSCLC) accounts for 80%∼85% of all lung cancers, and its 5-year survival rate is less than 15%, making the situation critical. In the past decades, despite some clinical advances in conventional treatments, the overall survival rate of NSCLC is still not optimistic due to its unique physiological conditions and the frequent occurrence of tumor escape. In recent years, immunotherapy has become a new hot spot in lung cancer research, including antibody therapy and cell therapy, which have been developed and utilized one after another, especially immune checkpoint inhibitor (ICI). These approaches have effectively improved the overall survival rate and objective response rate of NSCLC patients by enhancing the immune capacity of the body and targeting tumor cells more effectively, which is more specific and less toxic compared with conventional chemotherapy, and providing more strategies for NSCLC treatment. In this paper, we reviewed the relevant targets, clinical progress and adverse reaction in monoclonal antibodies, antibody-drug conjugates, ICI, bispecific antibodies, T-cell receptor engineered T cell therapy (TCR-T), Chimeric antigen receptor T-cell immunotherapy (CAR-T), and also report on their combination therapy from the immune-related background to provide better NSCLC treatment and prospective.

## Introduction

Lung cancer is the deadliest malignancy in the world with 2.2 million new cases and about 1.8 million deaths in 2020 (Sung et al., 2021). Non-small cell lung cancer (NSCLC) accounts for 80∼85% of the total number of lung cancers and is the main cause of mortality (Siegel et al., 2020). Unlike other cancers, smoking and secondhand smoke lead to a higher tumor mutational burden (TMB) in lung cancer, while the unique oxygen environment and pressure strengthen the role of molecular heterogeneity, and NSCLC is more likely to develop drug resistance, drug toxicity and cancer migration (Zito Marino et al., 2019). Clinically, only a small proportion of NSCLC patients are diagnosed at an early stage, and most of them are in the advanced or even metastatic stage, with a 5-year survival rate of less than 15% (Allemani et al., 2018). Conventional treatments for NSCLC are usually administered in the staged approaches, with complete surgical resection, usually a lobectomy, being recommended in the early stages (Saji et al., 2022), and stereotactic and hyper-fractionated radiation therapy may be considered for patients who are apprehensive about surgery (Tandberg et al., 2018; Chang et al., 2021; Dohopolski et al., 2021). Platinum-based combination chemotherapy and thoracic radiation therapy are commonly used for patients with advanced NSCLC (Vinod and Hau, 2020; Zhou et al., 2022). Percutaneous treatments such as thermal ablation as well as radiofrequency ablation can be used as a prognostic and maintenance treatment to the above treatments (Duma et al., 2019). Although these treatments have improved the survival rate of NSCLC patients, an important cause of death in lung cancer is due to the etiology or tendency of metastatic disease already present at the time of diagnosis and treatment, which cannot be effectively contained and treated by conventional means, suggesting that further survival improvement requires more effective approaches. The emerging immunotherapy in recent years has better potential to prevent cancer recurrence and metastasis by improving the patient’s own immune capacity and killing cancer cells specifically, raising the patient’s expectations for treatment. This article will discuss immunotherapy for NSCLC and related developments.

## Immune circulation and microenvironment in NSCLC

**FIGURE 1:**
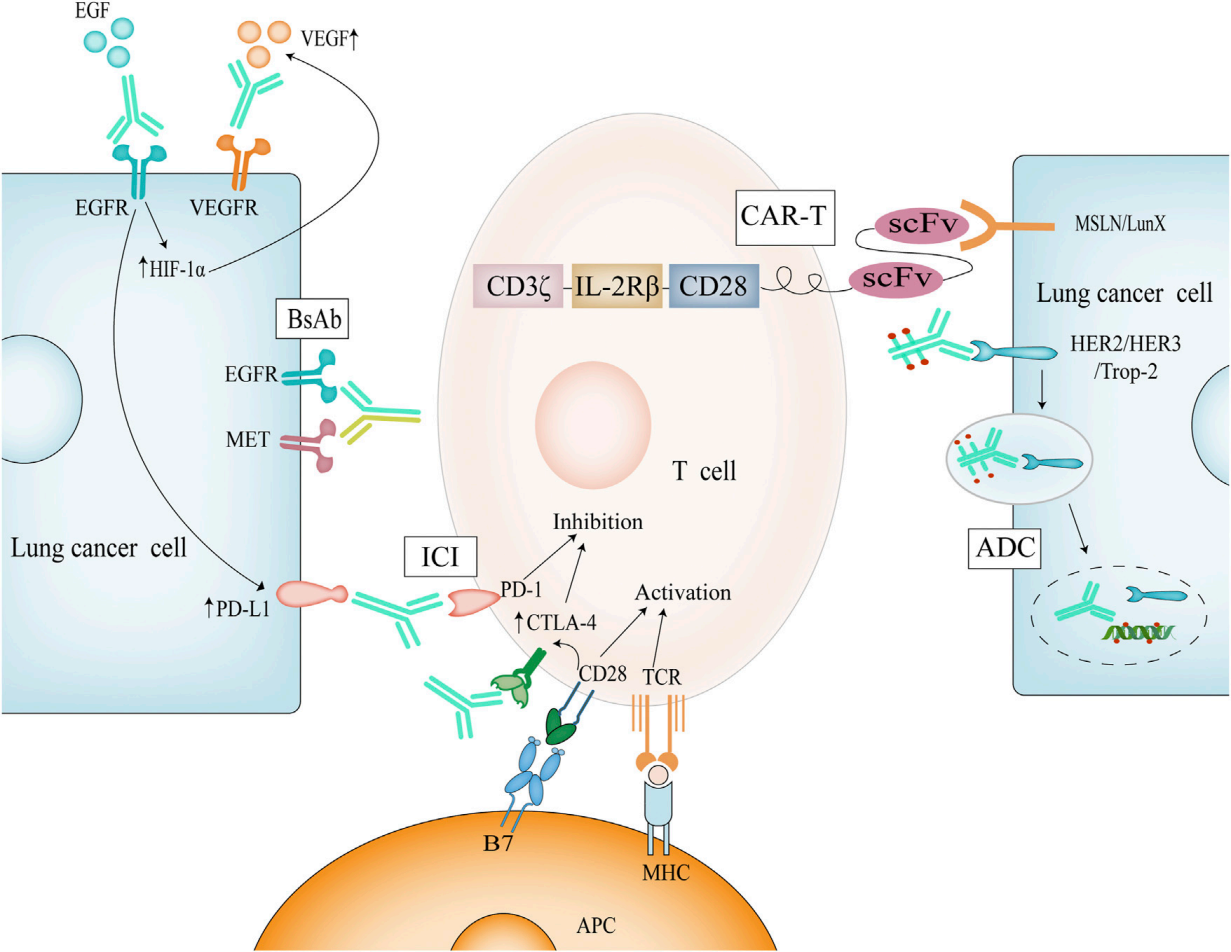
Mechanism of immunotherapy for NSCLC. Dual signaling activation of T cells causes upregulation of CTLA-4, which competes with B7 and thus regulates T cell activation. ICI can perform immune enhancement by blocking these targets; Activation of EGFR causes upregulation of HIF-1α and thus of VEGF. Simultaneous inhibition of EGFR and VEGF has a synergistic effect.

The body’s immune system is self-generated for the elimination of cancer cells and is a set of recyclable operations. The first is that cancer cells release the corresponding antigen, which is taken up by dendritic cells, and the antigen forms a complex with major histocompatibility complex (MHC) on the surface of dendritic cells; then dendritic cells present the antigen to T cells, when MHC and T cell receptor (TCR) bind, B7 protein on the surface of antigen-presenting cells binds to CD28 on the surface of T cells, and double signals stimulate T cell activation (Figure 1); Activated T cells come to the tumor tissue through the circulation and penetration; effector T cells specifically bind and kill cancer cells; dead cancer cells release more antigen to recruit more T cells (Chen and Mellman, 2013). Theoretically, this cycle will amplify the immune response, but in reality, the process does not occur as expected in cancer patients, because in order to prevent the occurrence of autoimmunity, the body’s multiple steps of this cycle are regulated and balanced in many aspects, and cancer cells use these regulations for immune escape. These phenomena occur during the antigen presentation stage, with down-regulation of MHC, decreased phagocytosis by IL-10-polarized macrophages, and release of more immunosuppressive factors (Pio et al., 2019); during the activation of T cells, cytotoxic T lymphocyte-associated antigen-4 (CTLA-4) competitively binds to B7 molecules and programmed death-ligand 1 (PD-L1) binding to programmed death-1 (PD-1) all inhibit T cells (Chen and Mellman, 2017); and during the infiltration stage of tumor tissue, vascular endothelial growth factor (VEGF) production of vascular structures and transforming growth factor-β expression all reduce invasion (Lee et al., 2020). By inhibiting the expression of the above molecules, it is the theoretical basis for immunotherapy of lung cancer.

The growth and characteristics of NSCLC are closely related to the tumor microenvironment (TME), which includes the non-immune microenvironment and the immune microenvironment, and their interactions influence the course of the disease and the role of therapy (Binnewies et al., 2018). The non-immune microenvironment includes extracellular matrix, fibroblasts, and the vascular system. Extracellular matrix and fibroblasts provide some structural help for tumor cells, and more importantly, it contains adhesion components, which physically affect the migration of tumor cells (Walker et al., 2018), while vascular endothelial cells will build a large number of new blood vessels at the tumor site and provide oxygen and nutrient substance for tumor development (Ferrara and Adamis, 2016); at the same time, tumor cells can secrete chemokines, which can recruit neutrophils and bind to them, and the process will not only promote angiogenesis but also may play a negative role in tumor prognosis (Zhang et al., 2019). The immune microenvironment is complex, including T lymphocytes, B lymphocytes, natural killer cells, macrophages and dendritic cells (Stankovic et al., 2018), among which regulatory T cells (Tregs) and related macrophages are more special and may produce immunosuppression during the development of NSCLC (Xie et al., 2020). Tregs, a subpopulation of T lymphocytes, express large amounts of CTLA-4 on their surface and can inhibit T cells by secreting suppressors or directly mediate the killing of effector cells. Under the action of chemokines, Tregs highly infiltrate around cancer cell tissues, that result in a strong immunosuppressive effect (Frankel et al., 2017). Tumor-associated macrophages, on the other hand, infiltrate near tumor cells through chemokines and produce polarization. Polarized macrophages express the immunosuppressive factor IL-10 as well as VEGF, resulting in vascular endothelial remodeling and concomitant migration (Wang et al., 2021). Therefore, the regulation of T cells, tumor-associated macrophages and the inhibition of VEGF could be an important means of treating cell lung cancer.

## Antibody therapy in NSCLC

### Monoclonal antibodies

Immunotherapy has been used in cancer therapy as early as the late 18th century (Loughlin and Coley, 2020), but there has been a lack of antibodies with high purity and specificity. In the late 20th century, hybridoma technology was born, which fuses B cells that specifically express antibodies with myeloma cells that can proliferate indefinitely *in vitro* (Parray et al., 2020). The monoclonal antibodies (mAbs) produced by hybridoma cells are of high purity and specificity, which greatly meet the needs of immunotherapy. In the last half century, mAbs have been developed rapidly, from the birth of hybridoma technology to the realization of fully human antibody technology, mAbs have become an important tool for cancer treatment (Waldmann, 2019).

Antibodies are usually composed of two identical light chains and two identical heavy chains linked by disulfide bonds. Due to the differences in heavy chains (α, δ, ε, γ and μ), antibodies can be classified into five categories: IgA, IgD, IgE, IgM and IgG. Among these five classes of antibodies, IgG accounts for 80% of human serum and almost all antibodies approved for use are IgG (Goulet and Atkins, 2020). IgG can be further divided into IgG1, IgG2, IgG3, and IgG4, which differ in the length of their hinge regions and the number of disulfide bonds, and more importantly, in their Fc functions. For example, IgG1 and IgG3 have a strong binding capacity to FcγR and can induce stronger antibody-dependent cell-mediated cytotoxicity (ADCC), while IgG2 and IgG4 have weak binding activity. Unlike other isoforms, IgG4 is completely unable to bind C1q to induce complement-dependent cytotoxicity (CDC). In addition, IgG3 has no binding ability to FcRn, resulting in a half-life that is only one-third that of the other isoforms (Yu et al., 2020). Therefore, it is crucial to select the appropriate antibody type according to the therapeutic need and the target of action.

In contrast to conventional therapies, antibodies not only kill cancer cells, but their Fab ends also bind to specific host targets to exert their corresponding effects.

### Immune checkpoint inhibitor

Immune checkpoint inhibitor (ICI) is a breakthrough therapy for NSCLC in recent years, and James P. Allison and Tasuku Honjo won the Nobel Prize in Medical Physiology as discoverers in 2018. ICI does not have a direct killing effect on cancer cells, but works by enhancing or restoring the patient’s autoimmune capacity (Li et al., 2019). Immune checkpoints are proteins on the surface of T cells that can negatively regulate immune switch and intensity, including CTLA-4 and PD-1 (Wei et al., 2018), whose purpose is supposed to control the immune response to protect the body from autoimmunity (Figure 1). Blockade of immune checkpoints relieves T cells of their braking signals, thus allowing T cells to continue their effects. Several ICIs have been used for NSCLC, but not every patient has responded well to them. The selection and detection of markers may play an important role, including biometric parameters of the tumor and relevant markers from blood sampling (Brueckl et al., 2020). In addition to combination with conventional chemotherapy, combination with stereotactic ablative radiotherapy may be a physiologically based approach, as radiation therapy suppresses the body’s proper immune response (Vansteenkiste et al., 2019).

### Antibody–drug conjugates

Most mAbs do not have antitumor activity in combination with antigen, even though they possess good specificity. Antibody-drug conjugates (ADCs) refer to the conjugation of antibodies to deliver highly toxic cargoes (payloads). Commonly used payloads today include calicheamicin and SN-38 against DNA and auristatins and maytansinoids against tubulins (Chau et al., 2019). Such antibodies with payloads specifically recognize and bind antigens and enter the cytosol by endocytosis, followed by the breakdown of the antigen-antibody-drug complex by a number of hydrolases and fibrinolytic enzymes, leading to the release of the drug intracellularly, thus providing a therapeutic effect (Hafeez et al., 2020) (Figure 1). In recent years, with the rapid development of ADCs, they have also shown good efficacy in the field of NSCLC. The main targets involved include human epidermal growth factor 2 (Her2), human epidermal growth factor 2 (Her3), and tumor-associated calcium signal transducer 2 (Trop-2), which are all in the clinical trial stage (Desai et al., 2022).

ADCs are a very successful form of drug delivery with both the specificity of immunotherapy and the powerful efficacy of chemotherapeutic agents, and their use can be foreseen in NSCLC. However, in the clinical phase of the drug, some adverse reactions due to the carriage of highly toxic substances have been identified. In the trastuzumab deruxtecan clinical trial, 19% of patients developed neutropenia and 26% developed associated lung disease to the point of death in two patients; patritumab deruxtecan may cause hematologic toxicity; and sacituzumab govitecan caused neutropenia in nearly 1/3 of patients (Heist et al., 2017; Jänne et al., 2022; Li et al., 2022). Therefore, it may be important to balance the relationship between antibodies and poisons, and the combination of other targets and other drugs may further improve the therapeutic effect.

### Antibody fragment

The development and utilization of antibody fragments may be a reliable way to address both efficacy and toxicity. Antibody fragments are classified as natural Fab and F (ab')2 and genetically engineered scFv, minibody, single-domain antibody, etc (Kholodenko et al., 2019). They retain the specificity to bind antigen, while the major difference from the common IgG-like mAbs is the absence of Fc structure. This leads to two advantages, on the one hand, a smaller molecular weight for better infiltration of the tumor microenvironment and passage through the blood-brain barrier, and on the other hand, the absence of Fc-mediated ADCC and CDC effects, which may be responsible for immunotoxicity (Chen et al., 2020). FDA has approved some antibody fragments to act in advanced acute lymphoblastic leukemia, North American rattlesnake venom infection, etc (Kantarjian et al., 2017; Cuker et al., 2019; Wilson et al., 2022). However, no antibody fragments have been approved for cancer therapy, including NSCLC. The structural advantages of antibody fragments cannot be ignored, and breakthroughs are being made with antibody fragment-drug conjugates (AFDCs) based on antibody fragments (Liu et al., 2019a; Jolivet et al., 2022), while clinical trials have been conducted for NSCLC (NCT01221675).

### Bispecific antibodies

The above mAbs have good specificity and are important tools for the therapy of NSCLC, but in the face of the complex TME and pathogenesis of NSCLC, a single target often fails to show satisfactory effects *in vivo* (Jänne et al., 2022), and bispecific antibodies can play a bridging or coordinating role against two epitopes in the immune microenvironment of NSCLC, showing great promise (Labrijn et al., 2019). At the same time, bispecific antibodies have better stability as well as lower side effects compared to co-formulation or simultaneous drug delivery because they are single molecules (Wang et al., 2019a). Bispecific antibodies can be broadly classified into two categories, one based on antibody fragment design and the other based on IgG-like design. Bispecific antibodies based on IgG-like design have cytotoxic presence and longer half-life due to the presence of Fc, but are prone to mismatching during production; in contrast, bispecific antibodies based on antibody fragment design have better tissue penetration and do not mismatch (Register et al., 2021).

## Targets

### Epidermal growth factor receptor

In NSCLC, epidermal growth factor receptor (EGFR) mutations as its important oncogenic pathway have been identified (Ye et al., 2021). The physiological role of EGFR is to regulate epithelial cell function (Sigismund et al., 2018), and mutated EGFR is aberrantly expressed to trigger cancer, and such mutations are found in more than half of Asian NSCLC patients (Lu et al., 2021). EGFR is a human epidermal growth factor receptor (HER) family receptor and a tyrosine kinase receptor (Liu et al., 2017). Correspondingly designed antibodies can block its ligand EGF from binding to it, thereby inhibiting EGFR phosphorylation and activation of downstream signaling for therapeutic purposes. Necitumumab is a fully humanized anti-EGFR mAb, which blocks its downstream pathway activation as well as dimerization by binding to the EGFR receptor (Thakur and Wozniak, 2017). Degradation of EGFR as well as ADCC effect can be clearly seen using necitumumab in NSCLC cells (Genova and Hirsch, 2016; Díaz-Serrano et al., 2019). Necitumumab in combination with chemotherapy can prolong survival by nearly 10 months in NSCLC patients with high EGFR expression (Thatcher et al., 2015) and has been approved as a first-line method for the treatment of advanced NSCLC (Table 1). However, it is inevitable that drug resistance develops after 10 months of medication, and some experiments have demonstrated that EGFR resistance achieves tumor escape by up-regulating PD-L1 expression (Figure 1) and inhibiting T cells (Peng et al., 2019). Therefore, the combination of Immune checkpoint inhibitors (ICIs) and EGFR mAbs may be a promising approach in the face of NSCLC patients with EGFR mutations that are high in PD-L1 expression (Yamada et al., 2019).

**TABLE 1:** Antibodies currently approved or in development for NSCLC.

Drug	Combination	Target	Study population	Stage	Toxicity	Reference
Necitumumab	with gemcitabine and cisplatin	EGFR	metastatic squamous NSCLC	FDA Approved (2015)	Skin rash, hypomagnesemia	Díaz-Serrano et al. (2019)
Bevacizumab	with carboplatin and paclitaxel	VEGF	non-squamous NSCLC	FDA Approved (2004)	Gastrointestinal perforation, pulmonary hemorrhage	Russo et al. (2017)
Ramucirumab	with docetaxel versus placebo and docetaxel	VEGF	Stage IV NSCLC after platinum-based therapy	FDA Approved (2014)	Fatigue, neutropenia	Garon et al. (2014)
Trastuzumab deruxtecan		HER2	metastatic HER2 mutation NSCLC	Clinical Phase II (NCT03505710)	Neutropenia, interstitial lung disease	Li et al. (2022)
Patritumab deruxtecan	with EGFR TKI	HER3	locally advanced or metastatic EGFR-mutated NSCLC	Clinical Phase I (U31402-A-U102)	hematologic toxicities	Jänne et al. (2022)
Sacituzumab govitecan		Trop-2	metastatic stage IV NSCLC	Clinical Phase I (NCT01631552)	Neutropenia, diarrhea	Heist et al. (2017)
Ipilimumab	with Nivolumab	CTLA-4	Advanced NSCLC	Clinical Phase Ⅲ (NCT02477826)	skin reactions, endocrine events	Hellmann et al. (2019)
Nivolumab	with Ipilimumab	PD-1	metastatic or recurrent NSCLC	FDA Approved (2015)	skin rash, hypothyroidism	Kazandjian et al. (2016)
Pembrolizumab	With pemetrexed and a platinum-based drug	PD-1	advanced NSCLC that lacks targetable mutations	FDA Approved (2015)	Pneumonitis, kidney injury	Kwok et al. (2016)
Atezolizumab	bevacizumab and chemotherapy	PD-L1	Metastatic Nonsquamous NSCLC	FDA Approved (2020)	Neutropenia, hypertension	Akinboro et al. (2022)
Durvalumab	Chemoradiotherapy	PD-L1	Unresectable stage III NSCLC	FDA Approved (2018)	pneumonitis	Mehra et al. (2021)
Amivantamab	with Platinum Chemotherapy	EGFR/MET	locally advanced or metastatic NSCLC harbouring EGFR Exon 20 insertion mutations	FDA Approved (2021)	Rash, paronychia	Syed et al. (2021)

Amivantamab, a bispecific antibody to EGFR and mesenchymal epithelial transition factor (MET), was approved for marketing by the FDA in 2021 for the treatment of NSCLC (Syed, 2021). MET is a proto-oncogene that encodes the hepatocyte growth factor receptor c-MET (Mo and Liu, 2017). Even though MET amplification occurs only in a small proportion of NSCLC, we still found that MET gene amplification and overexpression showed correlation with reduced degradation of c-MET as well as tumorigenesis (Drilon et al., 2017). Although EGFR inhibitors have shown good clinical results, it is noteworthy that their resistance is the biggest obstacle limiting their being used, and some studies have shown that MET amplification is an important mechanism for the development of their resistance (Wang et al., 2019b). There is new evidence that the combination of MET inhibitors with EGFR inhibitors is a promising therapeutic combination for the treatment of cancer caused by EGFR mutations in early and resistant stages of NSCLC (Pasquini and Giaccone, 2018). Amivantamab inhibits proliferation of tumor cell in NSCLC patients by effectively downregulating EGFR and MET gene levels and inducing immune antitumor activity and increasing IFNγ secretion (Yun et al., 2020). Among 81 patients with NSCLC after platinum-based chemotherapy, the overall remission rate was 40%, with a median duration of remission of 11.1 months and a median progression-free survival of 8.3 months (Park et al., 2021). Clinical studies have shown that amivantamab has a good and durable treatment effect, even its side effects such as rash and nail fungus accompany the treatment (Table 1).

### Vascular endothelial growth factor

Vascular endothelial growth factor (VEGF) is an important component of TME and plays an important role in regulating angiogenesis, and to inhibit its overexpression is an important strategy for the treatment of cancer (Frezzetti et al., 2017). VEGF belongs to the platelet-derived growth factor (PDGF) family, and its four types VEGF-A, VEGF-B, VEGF-C, and VEGF-D can activate downstream pathways by binding to three VEGF receptors (VEGFR-1, VEGFR-2, and VEGFR-3), leading to division and migration of endothelial cells, and the increase of vascular permeability (Melincovici et al., 2018). The growth of tumor cells is maintained by expressing VEGF to construct new blood vessels, which can supply more nutrients for themselves (Manzo et al., 2017), and mAbs play a therapeutic effect by blocking the binding of VEGF to receptors (Itatani et al., 2018). Currently, two mAbs against VEGF have been approved for the treatment of NSCLC, including bevacizumab against VEGF-A and ramucirumab against VEGFR-2 (Garcia et al., 2020) (Table 1). Compared with chemotherapy alone, the addition of bevacizumab prolonged overall survival by 2 months with fewer side effects, and based on this, bevacizumab was approved by the FDA for the first-line treatment of NSCLC in 2006 (Yamada et al., 2019). Ramucirumab also prolonged overall survival by 1–2 months and significantly increased response rate, and was approved for NSCLC treatment in 2014.

VEGF and EGF share a common downstream pathway, and activation of EGFR promotes the up-regulation of hypoxia-inducible factor 1 alpha (HIF-1α), further promoting VEGF expression (Nilsson et al., 2021), a process that is a positive feedback process (Figure 1). We observed VEGF up-regulation on EGFR-mutated tumor cells (Hung et al., 2016), and more importantly, EGFR is also expressed on tumor-associated endothelial cells. Based on the above, simultaneous inhibition of EGFR and VEGF may produce more powerful anti-tumor effects. Preclinically, it has been experimentally confirmed that the use of EGFR inhibitor bevacizumab in combination in models with EGFR mutations shows better antitumor activity and later acquired resistance (Masuda et al., 2017). In clinical trials, the progression-free survival of patients with advanced NSCLC treated with bevacizumab was prolonged by 3–7 months compared with erlotinib alone (Zhou et al., 2019; Maemondo et al., 2020), while the progression-free survival of patients with metastatic NSCLC treated with ramucirumab was prolonged by 7 months compared with erlotinib alone (Nakagawa et al., 2019). Based on the above data, the FDA has used ramucirumab in combination with erlotinib as a first-line option for the treatment of EGFR-mutant NSCLC.

### Human epidermal growth factor 2

Human epidermal growth factor 2 (HER2) is another proto-oncogene in the HER family. Unlike other receptors in its family, HER2 does not have a corresponding ligand to bind to it, and it exerts its activity by heterodimerizing with other EGFR receptors to promote cell growth and proliferation (Connell and Doherty, 2017). Overexpression of HER2 leads to abnormal cell growth and proliferation, which may be associated with the development of carcinogenesis, and HER2 overexpression is frequently seen in breast and gastric cancers, but less common in NSCLC patients, with an incidence of less than 5% (Takegawa and Yonesaka, 2017). However, following the use of EGFR inhibitors in NSCLC, amplification of the HER2 gene was unexpectedly found, which was importantly associated with resistance to EGFR inhibitors (Baraibar et al., 2020). To date, HER2-targeted therapy has not been approved for use in NSCLC patients. Trastuzumab deruxtecan consists of a humanized anti-HER2 monoclonal antibody, a cleavable tetrapeptide base linker, and a cytotoxic topoisomerase I inhibitor that prevents cancer cells from replicating DNA, leading to cancer cell death (Indini et al., 2021) (Table 1). Trastuzumab deruxtecan was approved by the FDA in 2019 for adult patients with unresectable or metastatic HER2-positive breast cancer (Keam, 2020). After the use of trastuzumab deruxtecan in patients with metastatic HER2-mutated NSCLC who were not responding to standard therapy, 55% of patients showed proven objective efficacy with a median duration of efficacy of 9.3 months, median progression-free survival of 8.2 months, and median overall survival of 17.8 months (Li et al., 2022). Therefore, treatment targeting HER2 may be one of the solutions for EGFR inhibitor resistance.

### Human epidermal growth factor 3

Human epidermal growth factor 3 (HER3) is a specific HER family member with little or no tyrosine kinase activity, and its activation depends on heterodimerization with another receptor, so it is generally not oncogenic when overexpressed alone (Haikala and Jänne, 2021). We can find abnormal HER3 expression in a variety of cancers, including breast cancer, prostate cancer, gastric cancer, and NSCLC, which may be associated with progression or poor prognosis in these cancers (Kawakami and Yonesaka, 2016; Scharpenseel et al., 2019; Gil et al., 2021). In EGFR-targeted therapy for NSCLC, there is experimental evidence that HER3 plays a key role in cancer cell survival and drug resistance (Yonesaka et al., 2016). However, as of now, there are no internationally approved therapies targeting HER3. Patritumab deruxtecan is an antibody-drug conjugate consisting of a HER3 antibody attached to a topoisomerase I inhibitor payload *via* a tetrapeptide-based cleavable linker for targeted delivery of cytotoxic drugs into cancer cells (Lim et al., 2022) (Table 1). In 57 patients with NSCLC previously treated with a tyrosine kinase inhibitor (TKI), the confirmed objective remission rate for patritumab deruxtecan was 39%; median progression-free survival was 8.2 months (Jänne et al., 2022). Since patritumab deruxtecan exhibits clinical activity to overcome EGFR TKI resistance mechanisms, we can expect that patritumab deruxtecan could be an option to overcome drug resistance in the future.

### Tumor-associated calcium signal transducer 2

Tumor-associated calcium signal transducer 2 (Trop-2), a transmembrane glycoprotein, is involved in intracellular calcium signaling (Goldenberg et al., 2018). Trop-2 is highly overexpressed in several solid tumors, especially in NSCLC, and its downstream signaling is involved in cancer cell survival, proliferation, migration and invasion (Shvartsur and Bonavida, 2015), which can be inhibited by knockdown of Trop-2 (Sun et al., 2020). Sacituzumab govitecan consists of a Trop-2 antibody coupled to a topoisomerase I inhibitor *via* a hydrolyzable junction (Bardia et al., 2021), which was approved by the FDA in 2021 for the treatment of refractory triple-negative metastatic breast cancer (Fleming et al., 2021) (Table 1). Among 54 patients with metastatic NSCLC treated with 10 mg/kg sacituzumab govitecan, an objective remission rate of 19% was achieved; the median duration of response was 6 months and the clinical benefit rate was 43%, indicating that the drug has a good durable response in patients with metastatic NSCLC (Heist et al., 2017).

ADCs are a very successful form of drug delivery with both the specificity of immunotherapy and the powerful efficacy of chemotherapeutic agents, and their use can be foreseen in NSCLC. However, in the clinical phase of the drug, some adverse reactions due to the carriage of highly toxic substances have been identified. In the trastuzumab deruxtecan clinical trial, 19% of patients developed neutropenia and 26% developed associated lung disease to the point of death in two patients; patritumab deruxtecan may cause hematologic toxicity; and sacituzumab govitecan caused neutropenia in nearly 1/3 of patients (Heist et al., 2017; Jänne et al., 2022; Li et al., 2022). Therefore, it may be important to balance the relationship between antibodies and poisons, and the combination of other targets and other drugs may further improve the therapeutic effect.

### Cytotoxic T-lymphocyte antigen 4

Cytotoxic T-lymphocyte antigen 4 (CTLA-4) is the first costimulatory receptor and have been found to play a role in immunosuppression. As mentioned previously in this article, T cell activation requires the binding of CD28 to B7, and when CD28 binds to B7 to activate T cells, it also promotes the expression of CTLA-4, which can compete with homologous CD28 to bind B7 ligands (Hosseini et al., 2020). The affinity of CTLA-4 to B7 is stronger than that of CD28, thus the expression of CTLA-4 inhibits the activation of T cells (Rowshanravan et al., 2018). Blocking the binding of CTLA-4 to its ligand allows more B7 ligands to bind to CD28 and achieve immune enhancement. Also, since CTLA-4 is highly expressed on the surface of Tregs, blocking CTLA-4 would reduce the immunosuppressive effect of Tregs (Tekguc et al., 2021). Until now, no CTLA-4 class antibodies have been approved for first-line NSCLC treatment alone, but it can be seen in combination with other drugs in the clinic. Ipilimumab is a fully human mAb against CTLA-4 that achieves anti-tumor effects by binding to CTLA-4 and blocking its action with the B7 molecule. In a clinical study, treatment of NSCLC patients with the combination of ipilimumab and nivolumab had an overall survival of 17.1 months, which was better than the overall survival of 13.9 months with chemotherapy alone (Table 1), and was independent of PD-L1 expression (Hellmann et al., 2019). However, one report showed that the combination of nivolumab and ipilimumab caused more immune-related adverse events than nivolumab alone (Shoushtari et al., 2018).

### Programmed cell death protein 1

Programmed cell death protein 1 (PD-1) is an innate immunosuppressive agent that has a small homologous sequence with CD28 and CTLA-4 and is expressed on B cells, T cells, dendritic cells, and NK cells, especially on the surface of T cells (Han et al., 2020). When T cells are activated, the second immune checkpoint PD-1 inhibits T cell activation and inflammatory factor production by binding to its ligands PD-L1 and PD-L2 leading to dephosphorylation of CD28, while cancer cells usually overexpress PD-L1 to escape (Ai et al., 2020). T cells that are in the TME for a long time will highly express PD-1 and show insufficient anti-tumor ability, and the immune ability of these T cells can be enhanced by blocking PD-1 (Jiang et al., 2019). PD-1 expressed on Tregs as well as tumor-associated macrophages, on the other hand, exerts an inhibitory effect on immunity and the anti-tumor effect can be improved by blocking it (Gordon et al., 2017; Overacre-Delgoffe et al., 2017). Nivolumab is the first PD-1 blocking mAb for the treatment of NSCLC, which significantly improves objective remission rate and overall survival compared with traditional chemotherapy (Borghaei et al., 2015), especially in patients with PD-L1 expression ≥50% in cancer cells, so nivolumab was approved by the FDA in 2015 for the treatment of metastatic NSCLC (Cortinovis et al., 2016) (Table 1). Pembrolizumab (Keytruda) is a humanized IgG4 anti-PD-1 mAb with no ADCC and CDC effects, and therefore is not cytotoxic. In a clinical study, not only did overall survival improve, but median progression-free survival was nearly 4 months longer with pembrolizumab administered in combination chemotherapy than chemotherapy alone (Gandhi et al., 2018). Pembrolizumab was approved by the FDA in 2015 as a first-line regimen for the treatment of metastatic NSCLC expressing PD-L1 (Table 1). However, after using pembrolizumab to treat lung cancer, some patients developed symptoms of colitis and pancreatitis (Ofuji et al., 2021).

### Programmed death-ligand 1

Programmed death-ligand 1 (PD-L1), the ligand of PD-1, belongs to the B7 protein series and is usually expressed on some macrophages and dendritic cells, and tumor cells tend to overexpress PD-L1 in order to escape from immune killing (Ohaegbulam et al., 2015). The expression of PD-L1 is regulated by interferon, and it has been experimentally demonstrated that IFN-γ leads to PD-L1 up-regulation in ovarian cancer cells (Abiko et al., 2015). It has also been shown that PD-L1 activates proliferation signals after binding to its receptor and non-immune proliferation occurs in tumor cells (Dong et al., 2018). Nearly 30% of NSCLC show high PD-L1 expression, so blocking PD-L1 can be effectively counteracted. Atezolizumab, a mAb that targets PD-L1 and activates T cells and kills tumor cells by blocking their binding to PD-1 (Crist and Balar, 2017), was approved by the FDA in 2016 for the treatment of NSCLC (Table 1). From clinical trials, atezolizumab monotherapy for NSCLC prolonged overall survival by nearly 7 months compared with chemotherapy alone, and the rate of adverse events was significantly lower than in the chemotherapy group (Herbst et al., 2020). In combination, atezolizumab used in addition to a VEGF inhibitor plus chemotherapy significantly improved overall survival as well as progression-free survival in patients with metastatic NSCLC (Socinski et al., 2018). Durvalumab is another PD-L1 antibody approved by the FDA for the treatment of NSCLC (Table 1). Statistically, durvalumab resulted in significantly better remission rates, median length of remission duration, and progression-free survival than the placebo group in a group of 713 NSCLC patients treated with radiotherapy and chemotherapy (Antonia et al., 2017), and after 4 years the durvalumab group still had a much higher survival rate of 49.6% than the 36.3% in the placebo group (Faivre-Finn et al., 2021), with the common malignant adverse effect being pneumonia.

## Cell therapy in NSCLC

Adoptive cell transfer therapy (ACT) is another general direction of immunotherapy for NSCLC, which is very personalized by directly infusing immune cells with anticancer activity into patients for treatment (Labanieh et al., 2018). One of its most important advantages is that the TME can be regulated by chemotherapy before immune cells can be infused back into the patient to provide support for the infused immune cells (Rosenberg and Restifo, 2015). Isolation of T cell populations with specific TCRs from the human body, massive expansion by T cell growth factor (IL-2) *in vitro* and finally reinfusion into patients to achieve cancer elimination are many ways for ACT application (Met et al., 2019). Clinical trials have shown that after using a combination of chemotherapy pretreatment, tumor infiltrating lymphocytes (TILs) and nivolumab, in 13 patients with advanced NSCLC, three patients experienced significant remission and 10 patients experienced symptom relief (Creelan et al., 2021). However, this process first requires resectable tumor tissue and the isolation of highly purified, specific T cells, a process that is time consuming and can lead to immune reactions due to purity (Sermer and Brentjens, 2019). With the development of genetic engineering technology, the insertion of exogenous TCR into T cells and chimeric antigen receptor (CAR) has become possible, and there is also good specificity when infused back into the body, and TCR-T and CAR-T technologies have developed rapidly (Chandran and Klebanoff, 2019; Chan et al., 2021).

### T cell receptor T cell therapy

The TCR in TCR-T, a dimer composed of α and β peptide chains, can specifically recognize and bind to MHC-presented intracellular and extracellular antigen fragments, thereby activating T cells to attack tumor cells (Zhao et al., 2021). However, natural TCR in the human body often have weak affinity for tumor cell antigens, and TCR-T relies on artificial coding to design high-affinity TCRs, which greatly improves the recognition affinity of T cells (Ohta et al., 2019). Data have shown that the affinity of amino acid-modified TCR for tumor cell common antigen TAA is significantly increased, and 80% of myeloma patients have good clinical inhibition performance (Rapoport et al., 2015). New York esophageal squamous cell carcinoma-1 (NY-ESO-1) is a cancer-testis antigen that is barely expressed in humans except in the testis, but is overexpressed in solid tumors (Thomas et al., 2018), and its overexpression may also cause spontaneous humoral and cellular immunity, making it a desirable therapeutic target (Table 2). Besides, NY-ESO-1 is also a good biomarker for anti-PD-1 treatment of NSCLC (Ohue et al., 2019). In a clinical study, NY-ESO-1 specific TCR engineered-T cells showed benefit in metastatic NSCLC (Xia et al., 2018).

**TABLE 2:** Clinical trials of cell therapy for NSCLC.

Type	Antigen	Study population	Stage	Toxicity	References
TCR-T	NY-ESO-1	metastatic NSCLC	Clinical Phase I (NCT02457650)	Transient anemia, White blood cell decrease	Xia et al. (2018)
CAR-T	EGFR	advanced relapsed/refractory EGFR-positive NSCLC	Clinical Phase I(NCT03182816)	Gastrointestinal perforation, pulmonary hemorrhage	Zhang et al. (2021)
CAR-T	LunX	NSCLC	preclinical		Hu et al. (2020)
CAR-T	PD-L1	NSCLC	Clinical Phase I (NCT03330834)	pulmonary	Liu et al. (2020)
CAR-T	Mesothelin	NSCLC	preclinical		Ye et al. (2019)

### Chimeric antigen receptor T cells

CAR-T are genetically engineered synthetic cells in which CARs can specifically recognize cancer cell surface antigens, and CARs consist of four components: extracellular antigen recognition domain, extracellular spacer domain, transmembrane domain, and intracellular T cell activation domain (Sterner and Sterner, 2021), and CAR-T cells can specifically recognize cancer cell surface antigens through single-chain variable fragment (scFv). T cells are activated by the signaling module CD3ζ and co-stimulatory molecules (CD28, 4-1BB) (Sadelain et al., 2017) (Figure 1). Dual-signal activation is the classical pathway of T-cell activation, in which the first generation of CAR-T cells rely only on CD3ζ to activate T cells, but T cells undergo rapid apoptosis (Zhang et al., 2017), the effect is not ideal; the second generation of CAR-T cells add co-stimulatory molecules CD28 or CD137, dual-signal activation amplifies the stimulation signal and promotes T cell proliferation (Lee et al., 2015); the third generation of CAR-T cells even contain two co-stimulatory molecules, further enhancing the ability to kill tumor cells; fourth-generation CAR-T cells increase the expression of cytokines, such as IL-2, which promotes T-cell growth and enhances T-cell activity. The fifth generation of CAR-T cells, perhaps due to their toxicity considerations, abandoned the design of dual co-stimulatory molecules that started in the third generation and added only the IL-2 receptor beta fragment to the second generation of CAR-T cells. Notably, Jonathan T Sockolosky et al. modified the amino acid site of murine-derived IL-2/IL-2Rβ and redesigned IL-2 to stimulate only T cells expressing orthologous IL-2Rβ, while wild-type IL-2 was unable to stimulate IL-2Rβ on T cells, avoiding systemic toxicity due to indiscriminate and unrestricted activation (Sockolosky et al., 2018).

Unlike TILs and TCRs, which specifically recognize MHC-presented antigens, CARs do not depend on MHC expression, and even if MHC expression is down-regulated in tumor cells, CARs still efficiently recognize antigens and kill tumor cells (Cohen, 2018), which may be the greatest advantage of CAR-T over other modalities. Of great significance, anti-CD19 CAR-T cells have been approved by the FDA for the treatment of hematological B-cell malignancies due to favorable clinical trials (Patel et al., 2020). In the study of CAR-T in solid tumors, a large part of it has focused on NSCLC (Kiesgen et al., 2018). Common targeted antigens of NSCLC include HER2, EGFR, PD-L1, Mesothelin, etc (Liu et al., 2020a; Qu et al., 2021; Zhang et al., 2021) (Table 2). Data support that after the use of EGFR-targeted CAR-T cells in 11 NSCLC patients, seven patients achieved response without malignant reaction (Feng et al., 2016). However, due to the high sensitivity of CAR-T cells, these antigens are also expressed in normal tissues in addition to tumor cells, which may lead to some side effects (Morello et al., 2016). Recently, lung-specific X (LunX)-CAR-T has been used to successfully eradicate NSCLC cells with high expression of LunX, and showed better infiltration (Hu et al., 2020). Unlike conventional targeted antigens, LunX is often highly expressed only in the lung and nose, but hardly in other parts of the human body, and cell therapy using LunX-CAR-T may be a new means of treating NSCLC. However, there are a number of factors that limit the development of CAR-T, starting with its extremely high cost of construction and labor, with the total cost of a complete treatment potentially reaching nearly a million dollars (Lin et al., 2018). During the course of treatment, cytokine release syndrome (CRS) and neurotoxicity have been identified, which must be given sufficient attention in future clinical studies (Brudno and Kochenderfer, 2019).

## Conclusion and perspectives

With the increasing understanding of physiological immunity in modern medicine, first-line clinical treatment options and preclinical studies for NSCLC have changed significantly from traditional surgical resection as well as systemic chemotherapy to targeted and personalized immunotherapy, highlighting the importance of immunotherapy for the improvement of solid tumors. We were surprised to see that these regimens were effective in improving overall survival and progression-free survival in NSCLC patients, and the FDA approved these drugs for marketing and as first-line treatment for NSCLC, but unfortunately these effects were not demonstrated in some patients, and accompanied by adverse effects and drug resistance. It is worthwhile to think about immunotherapy, and in order to further improve the efficacy, we need to have a deeper understanding of the mechanisms involved. We have detailed antibody therapy and cell therapy in immunotherapy, which includes first-line regimens and promising drugs still in clinical trials, but in the face of heterogeneous NSCLC, some patients are not suitable to use PD-L1 and EGFR expression profiles as a basis for clinical dosing regimen selection. Biomarkers play a key role in the selection of drug regimens, and the importance of accurately classifying therapies and developing additional biomarkers is now urgently needed.

EGFR mutations play a very important role in NSCLC, which are mainly exon 19 deletion mutations, exon 21 point-mutations, exon 20 mutations and exon 20 insertion mutations, etc (Harrison et al., 2020). Unfortunately, there are no corresponding antibodies for treatment except amivantamab, while small molecule EGFR TKI (tyrosine kinase inhibitor) has good clinical performance. Six EGFR TKI have been approved by the FDA: Gefitinib (Rawluk and Waller, 2018), Erlotinib (Abdelgalil et al., 2020), Afatinib (Harvey et al., 2020), Dacomitinib (Shirley, 2018), Osimertinib (Remon et al., 2018), and Mobocertinib (Markham, 2021). Further breakthroughs in the use of antibodies against EGFR mutations can be expected as research progresses.

In addition, Kirsten rat sarcoma (KRAS) is one of the most commonly mutated oncogenes, and its mutation is another very important cause of cancer in NSCLC, which accounts for a quarter of all oncogenic mutations (Liu et al., 2019b). Previous decades of research and treatment for KRAS have ended in failure, including, of course, antibody therapy and cellular therapy (Drosten and Barbacid, 2020). 2021 FDA approval of Sotorasib for metastatic NSCLC with KRAS G12C mutations suggests that more promising immunotherapy options are about to emerge (Reck et al., 2021).

In terms of regimen selection, in addition to the traditional combination with chemotherapeutic agents, we have found that some targets and pathways work better in combination, including EGFR with VEGF, PD-1 and CTLA-4, even though some of them have not been approved by the FDA for individual dosing. We can see them in some co-formulation as well as bispecific targets, but with improved efficacy, we should also be alert to their incremental toxicities.

In conclusion, although immunotherapy for NSCLC has made some achievements, more potential options are worth exploring at the same time. As theories and technologies become more sophisticated, better treatment options are being anticipated.
